# Adding Spice to the Slog: Humanities in Medical Training

**DOI:** 10.1371/journal.pmed.1001879

**Published:** 2015-09-29

**Authors:** Danielle Ofri

**Affiliations:** 1 Bellevue Hospital, New York, New York, United States of America; 2 New York University School of Medicine, New York, New York, United States of America

## Abstract

Writing from personal experience, physician and author Danielle Ofri asks what evidence is needed to justify trying to humanize medical training via the power of literature.

For years I dragged around poems in the pockets of my white coat, pressing them into the hands of unsuspecting medical students and residents. As an attending physician at a teaching hospital in New York City, my job was to supervise the medical students and residents. I had to ensure that our patients received good medical care and that our doctors in training were learning the ins and outs of inpatient and outpatient medicine.

But medical training is a stressful time. Conventional wisdom as well as a growing body of literature [1–3] suggests it is a critical component of the hardening of character, contributing to burnout, cynicism, brusqueness, and all the unappetizing traits we’ve come to hate in doctors. The humanities were one of those things that was thought might smooth some of the rough edges of training, humanizing—as the word itself suggests—our otherwise brutish selves.

American medical schools were giving at least lip service to the ideal of the well-rounded doctor, though not much was actually transpiring on the ground. Nobody was ceding academic territory from the basic sciences or clinical training to engage in the development of this much-lauded ideal.

Anyone foraying in this arena was pretty much left to squeeze it in on his or her own time, and on his or her own dime. For me, as I was testing the waters as a writer and also editing our homegrown literary journal, the *Bellevue Literary Review*, this came down to trying to slip in a few scattered specks of literature into the crevices of our hectic clinical and academic duties.

So, as soon as we’d finish rounds on the medical wards, I’d race to pass out an Anatole Broyard essay in the nanoseconds before dispersal entropy overtook our team of students and residents. I tried to condense our discussion of peptic ulcer disease in order to make time for a Chekhov story. I tried to slip in a William Carlos Williams poem between clinic patients.

I even bribed with food. Initially, I brought in cupcakes and sweets to go along with the literature. When I realized that my team hadn’t seen anything green in a month, I brought in fruit every morning from the Bengali fruit stand near the entrance to the hospital. I’d lay out my produce offerings next to my literary offerings in the doctors’ station and post a sign: “Fruit of the Day; Poem of the Day. Please Take One of Each.”

But no matter what I did, it always felt supremely awkward. Even though I knew—at least intellectually—that this was something good for my trainees, in the way that broccoli is good for the daily diet, I was always ill at ease. I could never tell whether my students and residents were drinking in this wholesome edification, whether they went along with it because any change of pace from the usual clinical slog was welcome, whether they were seething and resentful of the waste of their precious time, or whether it was something they benignly tolerated in order to placate a batty attending.

There seemed to be no way around the awkwardness. I’d briskly hand out a poem with an encouraging smile on my face, and then feet would shuffle and silence would ensue. No one would volunteer to read, so I would do so. Then more silence. More shuffling. Discreet glances at beepers, watches, and scut lists of the day’s tasks. So, then I’d offer a few observations, try my best to be witty and self-deprecating, and maybe offer a small clinical pearl that they might relate to.

The reciprocal gazes would be polite but imploring. It’s hard to hold court when your audience is begging for clemency, so I’d send them on their way, hoping that five minutes with Keats, Bellow, or Vonnegut would sink into their mental interstices, somehow bolstering them for the challenges that lay ahead.

My end-of-the-month evaluations tended to be trimodal. There was a small peak at “love the stories and poems,” but most centered around “cool stuff but takes away from medical learning.” And there was the third peak—“complete waste of time”—with the occasional “worst attending I’ve ever had.”

At some point, the efforts toward fighting the tide were just too taxing, and I gave up the formal distributions and discussions of literature. Medical humanities for me have become more desultory, though more spontaneous and perhaps more organic. When students—or patients, for that matter—notice the back issues of the *Bellevue Literary Review* spilling over on the filing cabinet of my exam room, I have the opportunity to wax enthusiastic about our institution’s literary efforts and press a few copies into their hands.

If a resident presents a case of prostatic hypertrophy and the resultant urinary symptoms, I can’t help but bring up Fermina Diaz’s observations about the natural history of male plumbing while overhearing her husband in the bathroom in *Love in the Time of Cholera*. On their wedding night, the “sound of his stallion’s stream” terrifies her, but decades later she shakes her head in disgust at the pathetic dribbles that splatter on the toilet seat.

I realize that my efforts are sporadic and are unlikely to effect large changes in our crop of future doctors. There is a trend, though, toward more formally incorporating humanities into the curriculum at many institutions, and stouter souls than me have been taking on the necessary turf battles.

Are there compelling reasons to be infusing the humanities into medical training? Well, there is some evidence that teaching the humanities helps buttress empathy [4–6], a crucial skill that can be in short supply (often because of the very nature of medical training). Medical humanities may also improve communication skills, deepen understandings of ethics, and ameliorate burnout—all hot-button issues.

Then there’s the notion that medical humanities are valuable because they might make medical students and doctors more interesting people to be with. For a cohort that mostly skipped English literature in favor of organic chemistry, this is something worth considering. Though there won’t be any placebo-controlled trials to prove this, I suspect that most patients appreciate their doctors’ ability to converse about something other than, say, their impending colonoscopy.

This is not to say that incorporating medical humanities is easy or even pleasant. All practical and logistical forces conspire against it. In a typical day in which an intern is required to stuff 37 hours of work into 16, even three minutes of poetry will feel punitive, and no amount of gustatory inducement can change that calculus.

Every medical trainee, down to the beginning medical student just signing for her first student loan, “knows” that the humanities are not part of the essentials for a real doctor—at best an extra, at worst fluff.

Of course, just because something falls into the category of fluff doesn’t mean it should be jettisoned. Whipped cream on hot chocolate may be wholly unnecessary fluff, but only the abstemious elect to do without. Ask any child—or any honest adult—and it becomes clear that whipped cream is an essential ingredient. The beleaguered medical humanitarians should embrace the so-called fluff. Fluff is what makes life tasty, interesting, and occasionally fun.

Try the following experiment: walk into the library and randomly select a student who has been blunt-force memorizing the inflammatory rheumatologic conditions. Offer that student the opportunity to elaborate on the distinguishing characteristics of the 20-odd vasculitides or the chance to tell the story of her most memorable patient experience. There may indeed be a few students who would prefer to expound upon eosinophilic granulomatosis with polyangiitis, formerly known as Churg Strauss disease, but it would likely be a minority.

Telling the story draws out our soul in a way that lists of diseases, however important, do not. Delving into character, setting, conflict, plot, and emotion offers a resonance that digs deeper and lasts longer than our mastery of medical knowledge. The humanities offer a framework for exploring these profound elements of medicine.

So, yes, I do believe that medical humanities are a critical component of medical education. And I do believe that they have unique and beneficial qualities that raise them above the level of other endeavors designed to make the medical training experience less onerous. Free meals in the hospital cafeteria, for example, would probably make our trainees happier, but I’m willing to stake the claim that it will not appreciably improve the character of our future doctors. Of course, I can’t say for sure that the humanities will, but I think the latter stands a better chance. And given that medical humanities won’t increase the waistline or cholesterol, I say, “Let’s go for it!”

Logistics dictates that the best we can achieve for teaching medical humanities are brief electives and individual lectures and workshops scattered throughout the training years. It’s legitimate to ask whether such tasting menus are even worth the effort. They will certainly never equal a classical undergraduate liberal arts education, but given that most of our medical trainees never experienced anything close to a comprehensive liberal arts experience, a brief exposure in medical school and residency may be the only points of humanities contact. In fact, it may be that medical trainees are more receptive to the humanities, given the parched arts background that most of us entered medicine with.

We need to accept the contradiction that humanities as an important part of training a doctor and also admit that teaching humanities in medicine is nearly impossible to do. Perhaps the only way this form of education will happen on a significant scale is if the top brass decides that the humanities are important and clears the way for it to take root.

There is precedence for such changes. Not even a generation ago, outpatient medicine was mainly an afterthought in most medical residencies. When I took my first office job right out of residency, my unsuspecting patients had no idea that I was more skilled at catheterizing their pulmonary arteries than in treating their sprained ankles. However, the tide was able to be changed, thanks to pressures from within, without, and above, and now outpatient medicine is a major pillar of medical residency. With the right pressures, humanities could likewise become an uncontested part of medical training.

When we look back at our medical education, and indeed our entire medical careers, which elements resonate most evocatively? What remains seared in our souls years and decades later? Most of us can’t recite from memory the differential diagnosis of palpable purpura and need a computer to distinguish renal tubular acidosis Types I, II, and IV (or even remember why there isn’t a Type III).

What we do remember, though, are the stories of our patients, in all their complicated, colorful, and chilling detail. If medical humanities can help us connect more with those stories, then let’s call in the humanities cavalry, even if there will never be a clinical trial to demonstrate clear and compelling benefit. Lots of what we measure in medicine is unimportant, and lots of what is important is unmeasurable.

When I began writing my book, *What Doctors Feel*: *How Emotions Affect the Practice of Medicine*, I sent out a call for stories. I asked people to send me stories of what made them the doctor they are today. I was flooded with responses, and not one person mentioned Harrison’s *Textbook of Internal Medicine*, *The Lancet*, or the disease formerly known as Churg-Strauss.

Instead, they related powerful stories of their patients. The skills they used to paint the pictures of these stories—interpretation, metaphor, character development, irony, connection, and perspective—were taken right out of the humanities playbook.

So, even if the evidence will never be as hard as the purists want, even if the humanities exposure will always be modest in scale and depth, even if there will always be a core of resistance from our learners, we should still sally forth armed with novel, poem, and painting. If one examined medical school critically—with all the years of training, all the endless memorization, all the loss of sleep, all the debt, all the misery, and the lack of evidence-based efficacy—no one would logically partake in it. Why should medical humanities be held to a different standard?
